# Effects of Contralateral Noise on the 20-Hz Auditory Steady State Response - Magnetoencephalography Study

**DOI:** 10.1371/journal.pone.0099457

**Published:** 2014-06-10

**Authors:** Hajime Usubuchi, Tetsuaki Kawase, Akitake Kanno, Izumi Yahata, Hiromitsu Miyazaki, Nobukazu Nakasato, Ryuta Kawashima, Yukio Katori

**Affiliations:** 1 Department of Otolaryngology-Head and Neck Surgery, Tohoku University Graduate School of Medicine, Sendai, Miyagi, Japan; 2 Laboratory of Rehabilitative Auditory Science, Tohoku University Graduate School of Biomedical Engineering, Sendai, Miyagi, Japan; 3 Department of Audiology, Tohoku University Graduate School of Medicine, Sendai, Miyagi, Japan; 4 Department of Functional Brain Imaging, Institute of Development, Aging and Cancer, Tohoku University, Sendai, Miyagi, Japan; 5 Department of Epileptology, Tohoku University Graduate School of Medicine, Sendai, Miyagi, Japan; 6 Department of Electromagnetic Neurophysiology, Smart Ageing International Research Center, Institute of Development, Aging and Cancer, Tohoku University, Sendai, Miyagi, Japan; University College of London - Institute of Neurology, United Kingdom

## Abstract

The auditory steady state response (ASSR) is an oscillatory brain response, which is phase locked to the rhythm of an auditory stimulus. ASSRs have been recorded in response to a wide frequency range of modulation and/or repetition, but the physiological features of the ASSRs are somewhat different depending on the modulation frequency. Recently, the 20-Hz ASSR has been emphasized in clinical examinations, especially in the area of psychiatry. However, little is known about the physiological properties of the 20-Hz ASSR, compared to those of the 40-Hz and 80-Hz ASSRs. The effects of contralateral noise on the ASSR are known to depend on the modulation frequency to evoke ASSR. However, the effects of contralateral noise on the 20-Hz ASSR are not known. Here we assessed the effects of contralateral white noise at a level of 70 dB SPL on the 20-Hz and 40-Hz ASSRs using a helmet-shaped magnetoencephalography system in 9 healthy volunteers (8 males and 1 female, mean age 31.2 years). The ASSRs were elicited by monaural 1000-Hz 5-s tone bursts amplitude-modulated at 20 and 39 Hz and presented at 80 dB SPL. Contralateral noise caused significant suppression of both the 20-Hz and 40-Hz ASSRs, although suppression was significantly smaller for the 20-Hz ASSRs than the 40-Hz ASSRs. Moreover, the greatest suppression of both 20-Hz and 40-Hz ASSRs occurred in the right hemisphere when stimuli were presented to the right ear with contralateral noise. The present study newly showed that 20-Hz ASSRs are suppressed by contralateral noise, which may be important both for characterization of the 20-Hz ASSR and for interpretation in clinical situations. Physicians must be aware that the 20-Hz ASSR is significantly suppressed by sound (e.g. masking noise or binaural stimulation) applied to the contralateral ear.

## Introduction

The auditory steady state response (ASSR) is an oscillatory brain response, which is phase locked to the rhythm of an auditory stimulus. ASSRs can be elicited by using repetition click, amplitude-modulated (AM) and frequency-modulated tones [1]–[4]. ASSRs have been recorded in response to a wide frequency range of modulation and/or repetition, but the physiological features of the ASSRs are somewhat different depending on the modulation frequency. Comparison of ASSRs generated by stimuli modulated with a frequency greater than 70–80 Hz (80-Hz ASSR) and ASSRs generated by stimuli modulated with a frequency around 40 Hz shows that the amplitude of the 40-Hz ASSR is significantly greater than that of the 80-Hz ASSR in the awake condition [1], [5]. However, the 40-Hz ASSR is very sensitive to the arousal state, and is greatly decreased under the conditions of sleep or general anesthesia, whereas the 80-Hz ASSR is not so affected by the arousal state [4], [6]–[8]. Moreover, the effects of contralateral noise on ASSRs are known to depend on the modulation frequencies [9]–[11]. Contralateral white noise at a level which does not cause a significant psychophysical threshold elevation results in remarkable suppression of the 40-Hz ASSR, but no significant effects were observed in the 80-Hz ASSR [9]–[11]. Since the same level of contralateral noise also does not cause any significant effects on the auditory brainstem response and N1 cortical response [9], [11], these suppressive effects seem to be a characteristic property of the 40-Hz ASSRs. However, the effects of contralateral noise on the ASSRs elicited by modulation frequencies other than 40 Hz and 80 Hz are not known.

Recently, in addition to 40-Hz and 80-Hz ASSRs, the 20-Hz ASSR has been investigated in the diagnosis of several psychiatric disorders, as different physiological properties have been observed for the 20-Hz and 40-Hz ASSRs in patients with bipolar disorder and/or schizophrenia. The 40-Hz ASSRs were significantly reduced, whereas the 20-Hz ASSRs showed no significant reduction in these patients [12]–[15]. These findings appear to indicate that the 40-Hz and 20-Hz ASSRs have different properties. The major source of 20-Hz ASSRs is thought to be the auditory cortex, as for 40-Hz ASSRs, but the amplitude of 20-Hz ASSRs is usually slightly smaller than that of 40-Hz ASSRs [12], [15]–[17]. However, other properties of 20-Hz ASSRs have not yet been clarified.

The present study compared the effects of contralateral noise on the 20-Hz ASSR and the 40-Hz ASSR in normal subjects to gain a better understanding of the physiological properties of the 20-Hz ASSR.

## Materials and Methods

### Subjects

This study included 9 normal volunteers, 8 males and 1 female aged 31.2+/−3.42 years (mean +/− standard deviation), without histories of auditory diseases or neurological disorders. Audiometry revealed that all subjects had hearing level thresholds of 20 dB or better for frequencies from 0.125–8 kHz. All subjects were right-handed with scores above +90 on the Edinburgh Handedness Inventory [18]. Written informed consent in accordance with ethical committee of Tohoku University Graduate School of Medicine and the Declaration of Helsinki (1991) was obtained from each subject. The present study was approved by the ethical committee of the Tohoku University Graduate School of Medicine. All parts of the present study were performed in accordance with the guidelines of the Declaration of Helsinki.

### Stimuli

The test stimulus to record the ASSRs consisted of 1000-Hz long tone bursts (duration 5 s, rise-fall time 1 ms) with 100% AM at 39 Hz and 20 Hz with an exponential modulation envelope [19] (resulting in the 40-Hz and 20-Hz ASSRs, respectively), which was produced using a digital signal processing platform (TDT System III, Tucker-Davis Technologies, Gainesville, FL) under the control of an IBM PC/AT computer. The inter-stimulus interval was 3 s. The sound pressure level of the AM tone was 80 dB SPL and was presented monaurally. Continuous noise (white noise) at a level of 70 dB SPL was applied to the ear not receiving the amplitude-modulated tone bursts, which is the same noise condition (type and presented level) used in our previous study to observe the contra-noise effects on the auditory cortical responses (40-Hz ASSR and N100m) [11]. The test stimuli (AM tone and tone bursts) and noise were presented to the subject through tube earphones (ER-3A, Etymotic Research, Elk Grove Village, IL).

### Recording and analysis

Magnetoencephalography (MEG) recording of auditory evoked fields used a 200-channel whole-head type axial gradiometer system (MEG vision PQA160C, Yokogawa Electric, Musashino, Tokyo, Japan) in a magnetically shielded room. The sensors consisted of first-order axial gradiometers with a baseline of 50 mm, with each coil of the gradiometers measuring 15.5 mm in diameter. The sensors were arranged in a uniform array over a helmet-shaped surface at the bottom of the dewar vessel. The centers of two adjacent coils were separated by a mean distance of 25 mm. The field sensitivity of the sensors (system noise) was 3 fT/Hz within the frequency range used in the study. Auditory evoked fields were recorded only in the awake state as confirmed by real-time MEG monitoring of the occipital alpha rhythm. The MEG signal was band-pass filtered between 0.16 Hz and 100 Hz, and sampled at 1000 Hz.

Coils were attached at 5 locations on the head surface. They acted as fiduciary points with respect to the landmarks (nasion and preauricular points) and the position of the head within the helmet by passing currents through the coils and measuring the magnetic fields. In addition to these fiduciary markers, the head shape of each participant was digitized using a three-dimensional digitizer (FastSCAN Cobra, Polhemus Inc., Colchester, VT) and co-registered with individual structural magnetic resonance (MR) images acquired using a 3T MR system (Achieva, Philips, Best, the Netherlands).

The responses to stimuli without and with contralateral noise were recorded alternately at least twice. MEG signals were recorded for at most 600 s during the presentation of AM tone bursts with/without contralateral noise, and later analyzed (offline) using the built-in software in the MEG system (MEG Laboratory, Yokogawa Electric) to obtain the ASSRs. Data epochs of 1 s in duration, starting at the onset of the trigger signal synchronized with a certain phase of the amplitude modulation, were extracted from the serial recorded data after filtering with a digital band-pass filter (35–45 Hz for 40-Hz ASSR, 17–23 Hz for 20-Hz ASSR) and averaged in the time domain. Epochs with signals exceeding 3 pT were rejected on a single-channel basis, so that about 3000–4500 epochs (mean  = 3580.2, standard deviation  = 289.1) were usually used in the averaging process. The location of the signal source was estimated for the amplitude maxima of the responses for each hemisphere using an equivalent current dipole (ECD) model with the best fit sphere for each subject's head. We used a single ECD model based on Sarvas law [20] in a spherical volume conductor for identifying the sources of the magnetic signals. Phase-lags were usually present between the amplitude maxima of the bilateral hemispheres, so the location of the signal source was separately analyzed in the right and left hemispheres. Dipole location and orientation of the ECDs were calculated for the amplitude maxima of the response using the data from channels from each hemisphere. ECDs with a goodness-of-fit value of 90% were accepted and the source was superimposed on the three-dimensional MR image of the individual subject using a MEG-MR image coordination integration system and the measured responses were verified to originate from the auditory cortex.

In the present study, the effects of contralateral noise on the power of ASSRs were analyzed by focusing on the channels of the maximum signals measured over each hemisphere. The power of the ASSR measured at these channels was quantified by fast Fourier transform spectrum analysis using the built-in software in the MEG system (applied window function: Hamming window, length of FFT: 1 sec, spectral resolution: 0.488 Hz). The measured power of the ASSR obtained under the same sound conditions of signal and contralateral noise were averaged, and the effects of contralateral noise on the ASSRs were analyzed separately for the right and left hemispheres.

Subjects were instructed to stay awake during recording (subjects usually watched silent movies during the measurements to prevent the need for artificial conversation with the subjects if they appeared to be sleepy) because the cortical ASSR tends to be significantly reduced during sleep [4], [5], [21].

## Results

**Figure 1:**
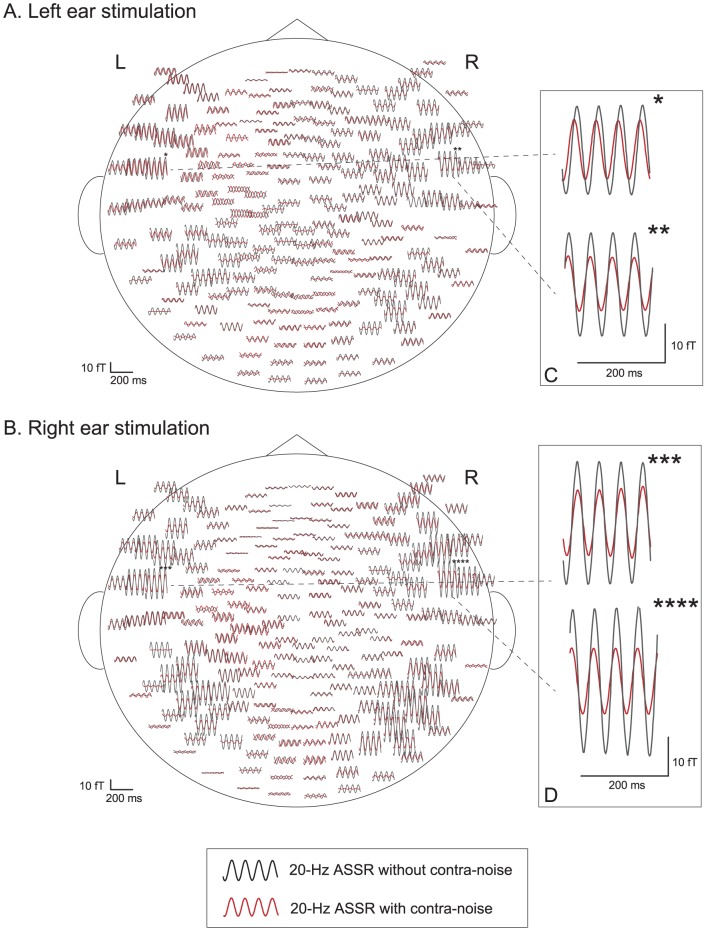
Example of the effects of contralateral noise on the waveform of the 20-Hz ASSR (Subject 3). A: left-ear stimulation with AM signal, B: right-ear stimulation with AM signal. ASSR with contralateral noise (red) are superimposed on waveforms without contralateral noise (black), mapped onto a flattened projection of the sensor array. Asterisks in A and B indicate the channels of maximum signals in each hemisphere. The responses of the channels of the maximum signals (indicated with asterisks) are magnified and shown in the inserts in the right column (C, D).

**Figure 2:**
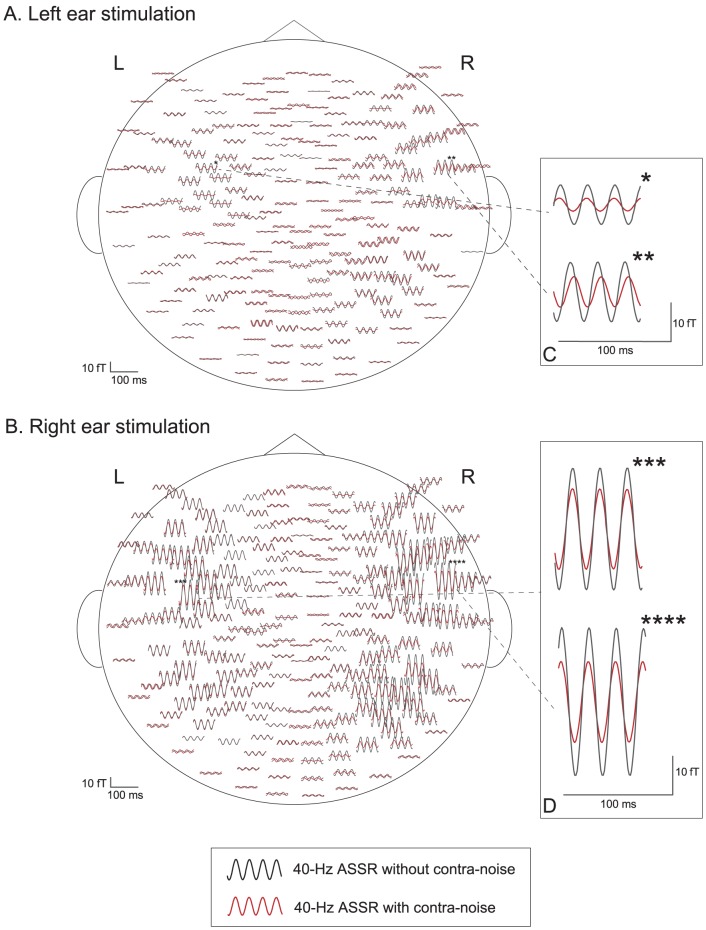
Example of the effects of contralateral noise on the waveform of the 40-Hz ASSR (Subject 3). A: left-ear stimulation with AM signal, B: right-ear stimulation with AM signal. ASSR with contralateral noise (red) are superimposed on waveforms without contralateral noise (black), mapped onto a flattened projection of the sensor array. Asterisks in A and B indicate the channels of maximum signals in each hemisphere. The responses of the channels of the maximum signals (indicated with asterisks) are magnified and shown in the inserts in the right column (C, D).

Both 20-Hz and 40-Hz ASSRs were observed bilaterally in all subjects under all stimulus conditions, using all combinations of right or left ear stimulation with or without contralateral noise. Figures 1 and 2 show examples of the effects of contralateral noise on the 20-Hz and 40-Hz ASSR waveforms, respectively, obtained from one subject (Case 3) mapped onto a flattened projection of the sensor position. Figures 1A and 2A show the responses to the stimulus and noise presented to the left ear, and Figures 1B and 2B show the responses to the stimulus and noise presented to the right ear. Figures 1C, 1D, 2C, and 2D illustrate the largest amplitude waveforms in each hemisphere. Both 20-Hz and 40-Hz ASSRs were suppressed by contralateral noise in the bilateral hemispheres, but the magnitude of suppression appeared to be larger in the right hemisphere than in the left hemisphere.

**Figure 3:**
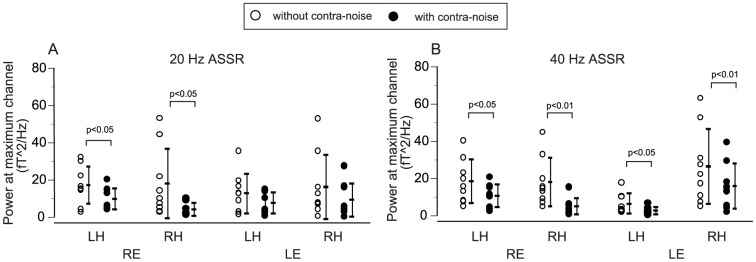
Effects of contralateral noise on the powers of ASSRs. The ASSR powers in the channels with the maximum responses were measured over each hemisphere for all measurement conditions (A: 20-Hz ASSR, B: 40-Hz ASSR) (see text for further details). LH: left hemisphere, RH: right hemisphere, RE: right ear, LE: left ear.

The effects of contralateral noise on the powers of ASSRs were analyzed focusing on the channels of the maximum signals measured over each hemisphere, as reported before [11]. Figures 3A and 3B show the averaged effects of contralateral noise on the power of the ASSRs in the channels of the maximum responses measured over each hemisphere for all measurement conditions. Data were statistically analyzed using three-way analysis of variance (ANOVA) with the factors of stimulation side (right/left ear), measured hemisphere (right/left), and presence of contralateral noise (off/on). Significant suppression of power was caused by contralateral noise for both 20-Hz and 40-Hz ASSRs, detected as significant main effects of hemisphere (p<0.01) and noise (p<0.001), as well as interaction between stimulation side and hemisphere (p<0.001), but the main effect of stimulation side was not significant.

**Figure 4:**
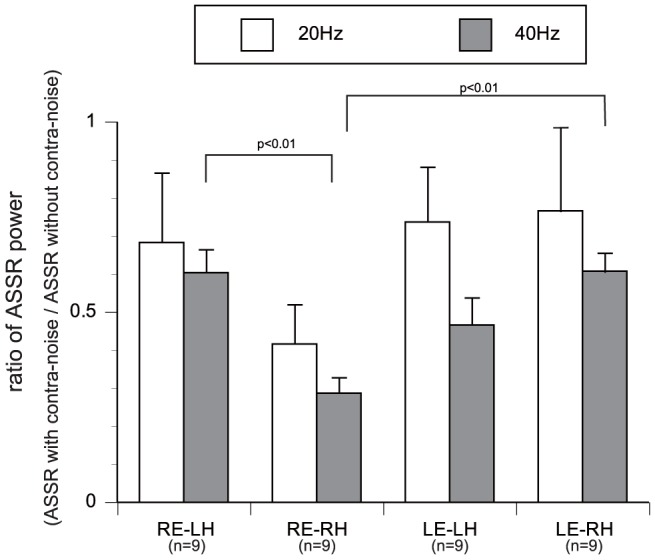
Ratios of ASSR power under conditions with/without contralateral noise. The average ratios of ASSR power are plotted with standard errors (error bars). Suppression of the 40-Hz ASSR was significantly greater than that of the 20-Hz ASSR (p<0.05) by three-way ANOVA (see text for further details). LH: left hemisphere, RH: right hemisphere, RE: right ear, LE: left ear.

The ratios of ASSR power between the conditions with/without contralateral noise, calculated based on the data shown in Figure 3, are plotted in Figure 4. Significant main effects of modulation frequency (p<0.05) and interaction between stimulation ear and hemisphere (p<0.05) were observed by three-way ANOVA with the factors of stimulation side (right/left ear), measured hemisphere (right/left), and modulation frequency (20 Hz/40 Hz). The magnitudes of contralateral noise suppression were significantly larger in 40-Hz ASSRs than in 20-Hz ASSRs. Moreover, the suppression was greatest in the right hemisphere with right ear stimulation.

## Discussion

In the present study, significant suppression of ASSRs caused by contralateral noise was observed in both the 20-Hz and 40-Hz ASSRs, although the magnitude of suppression was significantly smaller in the 20-Hz ASSR than in the 40-Hz ASSR. Moreover, the greatest suppression of the 20-Hz and 40-Hz ASSRs tended to occur in the right hemisphere during presentation of the stimulus to the right ear. This finding that the 20-Hz ASSR can be remarkably suppressed by contralateral noise is important to recognize.

ASSRs are thought to be generated throughout the central auditory system. However, the location of the highest activity depends on the modulation frequency [4], [22]–[24]. In general, when the ASSR is measured by conventional electroencephalography (EEG), the ASSR generated by stimuli modulated with a frequency around 80 Hz (80-Hz ASSR) contains more components from the brainstem [2], [4], [7], [23], [25], [26], whereas the ASSR generated by stimuli modulated with a frequency lower than 40–50 Hz contains more components from the upper auditory pathway [1], [4], [23], [27], [28].

On the other hand, the source reflected in the recorded ASSR is affected by the recording methods as well. Comparison of 20-Hz and 40-Hz ASSRs detected by EEG found that most components of the 20-Hz ASSRs originated from the auditory cortices, whereas the 40-Hz ASSRs may contain components from lower brain locations than the cortex such as the medial geniculate body in addition to components originating from the auditory cortices [23], [29], [30]. However, when the ASSR is measured by MEG as in the present study, almost all of the recorded ASSRs were assumed to consist of cortical components for both 20-Hz and 40-Hs ASSRs [31], [32].

The effects of contralateral noise on the ASSRs have so far been examined for the 20-Hz ASSRs by MEG (present study), 40-Hz ASSRs by EEG [9], [10] and MEG [11], [33], and 80-Hz ASSRs by EEG [10]. Contralateral suppression was only observed in the 20-Hz ASSR (present study) and the 40-Hz ASSR [9]–[11], [33], but not in the 80-Hz ASSR with EEG [10]. Considering that the 80-Hz ASSR recorded with EEG is thought to mainly consist of signals originating from the brainstem [2], [4], [7], [23], [25], [26], we suppose that contralateral noise might not so greatly affect the ASSR originating from the brainstem. On the other hand, contralateral noise suppression may be a phenomenon only observed in ASSRs originating from the auditory cortex. However, contralateral noise does not necessarily suppress other types of auditory cortical response such as N1 [11], [34], so this phenomenon may be quite specific to cortical ASSRs, and is presumably related to the auditory processing of the modulation.

Identification of the origins of 20-Hz and 40-Hz ASSRs with MEG has found no substantial difference between the locations of the cortical sources of the dipole moments of 20-Hz and 40-Hz ASSRs [12], [15]. However, a recent in vitro study of brain oscillation suggested that the origin of gamma-band oscillation might be different to that of beta-band oscillation [12], [35], [36], as the fast rhythmic bursting neurons in layer II/III and the pyramidal cells in layer V are closely related to the generation of the gamma-band and beta-band oscillations, respectively [35], [36]. These different sources for the gamma-band and beta-band oscillations may be one of the factors causing the observed differences between the 20-Hz and 40-Hz ASSRs measured with MEG, such as selective reduction of 40-Hz ASSRs in patients with bipolar disorder and/or schizophrenia [12]–[15].

Despite the probable differences in the physiological or pathophysiological properties between the 20-Hz and 40-Hz ASSRs, the temporal information regarding the modulation is likely to be analyzed in some common system, regardless of the modulation frequency. If so, the common features of ASSRs not depending on the modulation frequency, such as contralateral suppression of both 20-Hz and 40-Hz ASSRs, may reflect the general physiological properties of the common processing system for modulation in the cortex. Recently, the clinical importance of the 20-Hz ASSR has been emphasized, especially in psychiatry clinics, but the detailed physiological properties of 20-Hz ASSR are not so well known as those of the 40-Hz and 80-Hz ASSRs.

The present study newly showed that 20-Hz ASSRs are suppressed by contralateral noise, which may be important both for characterization of the 20-Hz ASSR and for interpretation in clinical situations. Physicians must be aware that the 20-Hz ASSR is significantly suppressed by sound (e.g. masking noise or binaural stimulation) applied to the contralateral ear. For example, masking noise is often applied to the contralateral ear to avoid cross talk effects in auditory examinations. The effects of this contralateral masking noise are likely to be negligible in measurements of the auditory brainstem response, N1 cortical response, and 80-Hz ASSR [9]–[11], [33], [34]. However, contralateral masking noise could significantly suppress the 20-Hz ASSR, as for the 40-Hz ASSR.
